# Drivers’ Intentions to Use Different Functionalities of Conditionally Automated Cars: A Survey Study of 18,631 Drivers from 17 Countries

**DOI:** 10.3390/ijerph182212054

**Published:** 2021-11-17

**Authors:** Tyron Louw, Ruth Madigan, Yee Mun Lee, Sina Nordhoff, Esko Lehtonen, Satu Innamaa, Fanny Malin, Afsane Bjorvatn, Natasha Merat

**Affiliations:** 1 Institute for Transport Studies, University of Leeds, University Road, Leeds LS2 9JT, UK; r.madigan@leeds.ac.uk (R.M.); Y.M.Lee@leeds.ac.uk (Y.M.L.); N.Merat@its.leeds.ac.uk (N.M.); 2 EICT GmbH, EUREF-Campus 13, 10829 Berlin, Germany; Sina.Nordhoff@eict.de; 3 VTT Technical Research Centre of Finland Ltd., P.O. Box 1000, FI-02044 Espoo, Finland; esko.lehtonen@vtt.fi (E.L.); Satu.Innamaa@vtt.fi (S.I.); Fanny.Malin@vtt.fi (F.M.); 4 SNF—Centre for Applied Research, Helleveien 30, NO-5045 Bergen, Norway; Afsane.Bjorvatn@snf.no

**Keywords:** automated driving, acceptance, survey, individual differences

## Abstract

A number of studies have investigated the acceptance of conditionally automated cars (CACs). However, in the future, CACs will comprise of several separate Automated Driving Functions (ADFs), which will allow the vehicle to operate in different Operational Design Domains (ODDs). Driving in different environments places differing demands on drivers. Yet, little research has focused on drivers’ intention to use different functions, and how this may vary by their age, gender, country of residence, and previous experience with Advanced Driving Assistance Systems (ADAS). Data from an online survey of 18,631 car drivers from 17 countries (8 European) was used in this study to investigate intention to use an ADF in one of four different ODDs: Motorways, Traffic Jams, Urban Roads, and Parking. Intention to use was high across all ADFs, but significantly higher for Parking than all others. Overall, intention to use was highest amongst respondents who were younger (<39), male, and had previous experience with ADAS. However, these trends varied widely across countries, and for the different ADFs. Respondents from countries with the lowest Gross Domestic Product (GDP) and highest road death rates had the highest intention to use all ADFs, while the opposite was found for countries with high GDP and low road death rates. These results suggest that development and deployment strategies for CACs may need to be tailored to different markets, to ensure uptake and safe use.

## 1. Introduction

Vehicles with automated driving features are slowly being introduced into the market. Widespread adoption of automated driving technologies has the potential to improve road safety, energy efficiency, and space utilisation [1,2,3], while also enhancing mobility for those unable to drive, such as older adults or people with disabilities [4]. Existing research has investigated potential future users’ a priori acceptance of these automated vehicles [5,6,7]. However, such research does not typically provide participants with detailed information about the functionality of the automated driving system under investigation. In addition, studies in this area usually focus on questions about scenarios far in the future, where fully autonomous vehicles can operate in all environments, without the aid of a human driver. For the near future, though, automated driving will be realised through individual Automated Driving Functions (ADFs), which manage specific operations, or manoeuvres, in specific scenarios, for example, changing lane on a motorway/highway. ADFs will be enabled by different technical systems (i.e., hardware and software), each with different Operational Design Domains (ODDs). SAE J3016 defines an ODD as “Operating conditions under which a given driving automation system or feature thereof is specifically designed to function, including, but not limited to, environmental, geographical, and time-of-day restrictions, and/or the requisite presence or absence of certain traffic or roadway characteristics” [8]. For example, a Motorway ADF would operate on motorways, and other two- and three-carriageway roads, in uncongested conditions at high speeds. A Traffic Jam ADF would only operate in high-density traffic, or congested motorways, and other two- and three-carriageways, at low to medium speeds, while an Urban ADF, would operate on urban street networks, at low speeds. Finally, a Parking ADF would perform parking manoeuvres in a closed environment, such as a private parking lot, or designated area.

Based on SAE J3016, a vehicle with ADF(s) that perform the dynamic driving task, but require the driver to respond to operate the vehicle when something goes wrong, can be considered Level 3, or as having “Conditional Driving Automation” [8]. For the purposes of this paper, we refer to these vehicles as Conditionally Automated Vehicles (CACs). In the text below, we also use the term Automated Vehicles (AVs) to refer to research that has used this or other generic terms such as “driverless vehicles”, “autonomous vehicles”, or “self-driving cars”.

ADFs are distinct from one another, not only in terms of their technical capabilities (e.g., performing individual driving subtasks on certain road types, in certain weather conditions), but also regarding what benefits they may offer to drivers. Research shows that there are differences in terms of users’ acceptance of different Advanced Driver Assistance Systems (ADAS; e.g., Adaptive Cruise Control or Park Assist). Therefore, it follows that there also might be differences between ADFs in terms of how they are perceived and accepted by drivers [9]. However, there is currently a lack of understanding of user acceptance of different ADFs, and whether this is affected by individual differences, for example, based on age, gender, experience with different, and country of residence. Understanding what functionalities consumers want from their vehicles is especially important if the anticipated benefits of automation are to be realised through the mass adoption of this technology by a wide range of customers across the globe. Therefore, there needs to be a solid evidence base that guides developers of ADFs and accelerates their deployment by different markets.

This study uses an online survey, administered to 18,631 drivers from 17 countries, to assess how drivers’ intention to use ADFs in motorway, traffic jam, urban, and parking environments varies based on their individual differences, and whether this is influenced by a country’s socio-economic status and road safety statistics. According to Warshaw and Davis [10], the psychological construct “intention to use” can be defined as “the degree to which a person has formulated conscious plans to perform or not to perform some future behaviour” ([10], p. 214). In the context of this paper, intention to use can be defined as “the degree to which a person has formulated conscious plans to use or not to use an automated driving function in the future”. The construct “intention to use” forms part of all models of seeking to explain factors that influence users’ behavioural intentions to adopt technology. For example, intention to use is the primary outcome variable of the unified theory of acceptance and use of technology (UTAUT) model [11], which integrates core elements from eight models and prominent theories (including the theory of reasoned action (TRA), innovation diffusion theory (IDT), the theory of planned behaviour (TPB), the technology acceptance model (TAM), the combined TAM-TPB, the motivational model (MM), the model of PC utilization (MPCU), and social cognitive theory (SCT)) to predict and explain new technology adoption, acceptance, and usage. UTAUT has been used extensively in applied research, and recently a number of papers have used UTAUT to understand the factors influencing users’ behavioural intentions to adopt automated vehicles [5,12]. Therefore, the intention to use construct will be used in this study to focus on users’ behavioural intentions to adopt ADFs.

We begin with a review of the literature around the intention to use Conditionally Automated Cars (CACs), focusing on the effect of individual differences, and country of residence, followed by the results of our survey study, including an overview of the trends across individuals and countries. Finally, we discuss how the differences in intention to use ADFs, found in this study, may inform policies and strategies around the development, deployment, and marketing of these systems, and how such decisions are likely to be influenced by the disparity in socio-economic and road safety statistics of low and high Gross Domestic Product (GDP) countries.

### 1.1. Influence of Age and Gender on Intention to Use CACs

To date, research in this context has found an inconsistent effect of age and/or gender on intention to use automated vehicles. For example, numerous studies have reported that, compared to older individuals (e.g., >50 years), younger individuals express higher rates of acceptance of driverless cars [4,13,14,15,16]. Zmud and Sener [17] suggest that this may be linked to the desire for this demographic to be more productive while travelling, which includes engaging in work-related tasks, social media, shopping, or generally a desire to interact more with mobile technologies. Regarding gender differences, male respondents report more favourable attitudes towards automated vehicles than [18,19], which is unsurprising, as males are typically reported to be more inclined than women to use technology [20]. Moreover, males tend to express less concern about automation failures, and are more anxious about liability issues. On the other hand, females are more worried about losing control of the vehicle [21]. However, these findings are not particularly helpful for understanding drivers’ intention to use specific ADFs, as participants are often asked about their acceptance of the entire system, rather than specific functionalities, or use cases. Indeed, it can be argued that, there are some situations where females are likely to feel more receptive to automated vehicles than males, and where older individuals will prefer automation more than younger individuals. In a series of focus groups conducted by [22], females were more open to fully driverless cars than men, as they said they would have more time to take care of their children in the back seat. Several studies have shown that individuals exhibit greater intention to use driverless cars with increasing age [19,23,24,25], with some authors suggesting that age differences in intentions to use may be based on different perceptions of when and where automation might be useful. This has recently been shown in a large-scale consumer survey study by [26], who report that younger respondents were initially more willing to use a self-driving car compared to older respondents. However, when respondents were asked if they would be willing use these vehicles when they were no longer able to drive, or if they were assured that it drove as safely as them, there were no differences between age groups. There is some evidence of this trend in the literature on gender and age differences in the acceptance and use of ADAS. While these examples are not intended to promote gender or age stereotypes, they do demonstrate the importance of situation-specific assessment of intention to use CACs.

There is little research on gender and age differences regarding individual ADF use. However, since ADFs can be considered an evolution of ADAS, it may be informative to consider the literature on gender and age differences in acceptance and use of ADAS. A survey of ACC owners, conducted by [27], found that only 6% of the older (>65 years) male respondents did not know how to use the system, compared to 35% of older (>65 years) female respondents. Some of these differences between males and females are thought to be due to differences in travel preferences and opportunities, for example, in terms of mode choice, travel time, trip purpose, trip route, and trip chain [28]. For example, traditionally, females travel less often, and for shorter distances, than males [29]. They also make fewer journeys to work by car, and more journeys for shopping and care-giving duties [30,31]. It can be argued, therefore, that based on these travel patterns, females will have a greater intention to use ADFs in urban and parking environments, while males might have greater intention to use ADFs in motorway and traffic jam settings. However, many of these differences in travel pattern are attributed to the gendered division of work in households. Although the travel behaviour of males and females may eventually converge, as more females enter the workforce in a global context, the gender gap in low-income countries is deeply entrenched [28]. Therefore, it is important to assess whether the intention to use specific ADFs is governed by gender, as this may help city planners, lawmakers, transport operators, and OEMs develop more efficient and equitable transport policies and technologies by considering the different users’ needs.

### 1.2. Influence of ADAS Experience on Intention to Use CACs

A factor not considered in many studies on acceptance of CACs is how previous ADAS experience influences users’ views of new, more advanced versions of these technologies. As argued above, ADFs are an evolution of ADAS, so it is important to understand whether intention to use an ADF is influenced purely by its technical capabilities, or whether drivers’ previous experience with ADAS also plays a role in this context. There is some evidence that previous ADAS use may have a positive effect on intention to use CACs. For example, the authors in [24] found that drivers with experience of various ADAS (e.g., parking assist and cruise control) were more likely to accept an autonomous driving feature in their car than those with little or no experience of these systems. However, the authors noted that there was a low ownership rate of ADAS systems amongst their sample (<20%). Similarly, the authors in [32] showed that drivers who had had in-vehicle technologies in their car (e.g., lane departure warning, adaptive cruise control), were more likely to accept higher levels of automation. However, these authors defined automation in their study in terms of the SAE [8] levels, rather than in terms of specific system functionalities. It can be argued that it is useful for car manufacturers to understand whether owning a parking ADAS, for example, increases a consumer’s likelihood of using a parking ADF, and, how experience with one type of ADAS is linked with intentions to use other types of ADFs. This, in conjunction with an understanding of the needs and desires of individuals in different markets, car manufacturers can use to develop more targeted ADF development and marketing strategies.

### 1.3. Influence of Country on Intention to Use CACs

To date, low-income countries (as defined by Gross Domestic Product) have been under-represented in research on CACs. However, as these countries are typically over-represented in terms of road-related deaths and injuries [33], they will benefit immensely from the widespread deployment of ADAS, and CACs, to help improve road safety. Studies suggest that there are currently differences across the globe regarding when, and how, CACs will be deployed. For example, in [34], it is predicted that Europe will lead the world in terms of penetration of automated driving, with up to 4.9% of new vehicles expected to have autonomous driving features by 2023, while in South America and Africa, this figure is expected to be only around 0.4% and 0.1%, respectively. Differences in estimated penetration rates are reflected by current, and forthcoming, regional differences in the socio-economic, infrastructural, and legal foundations required to deploy CACs. For example, for CACs to operate effectively, they require high-definition mapping and high-quality road infrastructure, which are more prevalent in high-income countries. However, even if the technical and legal foundations are in place, CACs will only succeed once they have been accepted by the public.

Previous research has shown that acceptance of AVs tends to be higher in lower-GDP countries [35,36], and vice versa. However, these studies typically ask respondents to give their opinions about using automated driving systems, with little or no focus on particular use cases, or a description of system capabilities. Moreover, very few studies have investigated the effect of age, gender, previous experience with ADAS, and country of residence on acceptance and intention to us, making it challenging to obtain a detailed understanding of the factors that influence any country-related differences. Insights into such variations in intention to use across a range of factors are important, as they will provide a better understanding of user needs and preferences, and more accurate estimation of the likely penetration rate of different ADFs into the market. This knowledge will also help developers in the design of future systems and provide more accurate assessments of the benefit of such technologies for policy makers and planners.

### 1.4. Research Objectives

To understand these issues further, the aim of this survey-based study, conducted as part of the L3Pilot project, funded by the European Commission, was to investigate drivers’ intention to use ADFs in four different ODDs: Motorways, Traffic Jam, Urban, and Parking, and understand whether these intentions were influenced by age, gender, previous experience with ADAS, and respondents’ country of residence. We addressed the following research questions:

1. Does intention to use CACs differ across different ODDs as defined by Motorway, Traffic Jam, Urban, and Parking environments?
2. How does intention to use CACs in different ODDs vary by age and gender?
3. Or does intention to use CACs in different ODDs vary based on respondents’ experience with ADAS?
4. Are these responses different across different countries?

## 2. Methods

### 2.1. Questionnaire Design and Content

The primary aim of the survey was to investigate the acceptance of CACs among car drivers. The questionnaire contained questions about respondents’ travel behaviour, their socio-demographic characteristics and familiarity with ADAS, their understanding of the concept of CACs, and attitudes towards CACs, including the extent to which respondents felt their intended use of CACs might affect their future travel behaviour, in terms of travel mode, trip type, and route choice.

At the start of the survey, respondents were given descriptive information about the functionality of the CACs, as follows:

*“Conditionally automated cars can drive under limited conditions, such as driving on motorways, on congested motorways, in urban traffic, and in parking situations. They will not operate beyond these conditions.”*

*“Conditionally automated cars do the steering, acceleration and braking. They will stay in the lane and maintain a safe distance to the vehicle in front. They will also overtake slower moving vehicles or change lane. These cars still have gas and brake pedals and a steering wheel.”*

*“You are not driving when the car is in conditionally automated mode—even if you are seated in the driver’s seat. This will allow you to engage in other activities, such as emailing or watching videos. However, the car might ask you to resume vehicle control anytime, e.g., when approaching a construction site, which means you might have to stop what you are doing and resume control of the car.”*

After answering a series of comprehension questions about CACs based on the text above, respondents from each country were randomly split into four equally sized sub-groups, balanced by age and gender. Each sub-group answered questions about using one of four ADFs that were operational in Motorway, Traffic Jam, Urban, or Parking environments. The sample was split in this way, to reduce the total time needed by each participant to complete the survey. Before answering these questions, respondents were provided with descriptions of the functionality of each ADF (see Table 1).

The aim of the current study was to assess the responses to questions on the intention to use ADFs. The questions were developed using the “behavioural intention” construct of the Unified Theory of Acceptance and Use of Technology (UTAUT) [11,37]. The intention to use ADFs was measured by one item, which was, “I plan to use a conditionally automated [ADF type] once it is available”. The wording of the statement was modified to reflect the different ADFs. See Table 1 for the questions for each ADFs.

Respondents’ level of agreement with the statements on intention to use was rated on a five-point scale, where 1 = Strongly disagree; 2 = Disagree; 3 = Neutral; 4 = Agree; and 5 = Strongly agree. We calculated the mean intention to use score, with higher scores indicating a greater agreement with the questionnaire item.

As described in the Introduction, the present study investigated whether experience with advanced driver assistance systems (ADAS) influenced respondents’ intention to use CACs in different ODDs. Respondents were asked to indicate their experience with several ADAS. Initially, we assessed their response based on the original categories given (see Table 2). However, to simplify the analysis, we re-coded the responses for those who reported having the respective ADAS, and those who reported either not having, or not knowing whether they have, a particular ADAS. Respondents were given a brief description of the ADAS before indicating their experience with it (see Table 3), to ensure they understood which ADAS they were being asked about.

### 2.2. Procedure and Recruitment

The questionnaire was translated by a translation bureau into the national or predominant language of the respective countries and administered by the German market research institute INNOFACT AG (www.innofact.com), using the survey tool EXAVO (www.exavo.de/surveytainment/). The only exception here was Finland, where the questionnaire was translated by Finnish project partners, and data collection was conducted by Taloustutkimus Oy (www.taloustutkimus.fi), using a nationally representative Internet panel. Data were collected between April and June 2019 (UK, Finland, Sweden, Germany, Italy, France, Hungary, China, USA) and in March 2020 (Spain, Brazil, India, Indonesia, Japan, Turkey, South Africa, Russia).

Prior to the survey launch, the questionnaire was pre-tested among the project partners, using several iterations, to ensure that there was a logical ordering and precise meaning of the questionnaire items. A soft launch of the questionnaire was then performed with thirty respondents, before the official launch, to resolve any implementation or wording errors.

The survey was administered online to 18,631 respondents from 17 countries, covering all inhabited continents. Table 4 shows the sample size for each country, broken down by age group and gender, which were selected to investigate the effect of differences in attitudes towards CACs. The selection of countries was based on the current, or projected, strength of their car market. We sought to have at least one country from each continent.

The invitation to participate in the survey study was advertised via online research panels, which have access to a large number of respondents, by email. Respondents received between 0.80 and 1.00 Euro upon completion of the survey, which could be redeemed as vouchers. Respondents in Finland had a chance to win prizes.

The sample only included current car drivers, as respondents were excluded from the dataset if they indicated that they “almost never” used a private car, car-sharing, or rental car as a driver. We focused on existing car drivers, as they represent the cohort of potential future users of CACs and the example ADFs under investigation in this study.

### 2.3. Data Filtering

Data were filtered for missing data. Respondents were also excluded if they indicated that they used all transport modes daily and/or indicated that they make daily use of an aeroplane, as these atypical travel behaviours. In order to select car drivers into the sample, respondents were removed from the data if they reported to always never used the private car as driver (without car-sharing and rental cars) and the car as driver (only carsharing and rental cars) or replied to these two questions with “I prefer not to respond”. Furthermore, “I prefer not to respond” answers were defined as missing values and excluded from the analysis. Respondents were also excluded if they provided inconsistent or unlikely responses to the socio-demographic questions (i.e., being 20 years old and retired), and/or responded “I don’t know” to all questions measuring the knowledge of the description of conditionally automated cars. At the end of the second stage of the data filtering, 18,631 questionnaires were retained for analysis.

### 2.4. Data Analysis

Statistical analysis was performed using SPSS version 16 software. Except for an initial comparison of the ADFs, data for each ADF was analysed separately, as they were provided by independent groups. The tests for normality, examining skewness, and the Shapiro–Wilk tests for group-level differences, indicated the data were not normally distributed. Moreover, Levene’s F tests revealed that the homogeneity of variance assumption was not met (*p* < 0.05). As such, the Welch’s F and Games–Howell post hoc tests were used, as they do not require the groups to have equal standard deviations. An alpha level of 0.05 was used for all subsequent analyses. Omega squared (ω^2^) was calculated as a measure of effect size, and is an estimate of how much of the variance in the response measures (intention to use) is accounted for by the explanatory variables (age, gender, experience with ADAS, and country of residence).

## 3. Results and Discussion

### 3.1. Intention to Use Different ADFs

To compare the effect of ADF type (Motorway, Traffic Jam, Urban Road, and Parking) on intention to use scores, we conducted a one-way, between-participant Analysis of Variance (ANOVA). Results showed a significant effect of ADF type on intention to use scores (*Welch’s F* (3, 10,280.035) = 76.832, *p* < 0.001). The estimated omega squared (ω^2^ = 0.01) indicated that only 1% of the total variation in the average intention to use score was attributable to differences between the ADFs. Post-hoc comparisons, using the Games–Howell post hoc procedure, were conducted, to determine which pairs of the four ADF means differed significantly from each other. Results showed that the mean intention to use score for the Parking ADF (M = 3.70, SD = 1.09, *n* = 4635) was significantly higher than the Motorway ADF (M = 3.44, SD = 1.18, *n* = 4629), Traffic Jam ADF (M = 3.40, SD = 1.17, *n* = 4622), and Urban Road ADF (M = 3.40, SD = 1.18, *n* = 4632), but there were no differences between the other ADFs.

This result is perhaps not that surprising, as parking a vehicle is fundamentally different to controlling it on a motorway, or operating it during a traffic jam, or on urban roads. The latter three all require the maintenance of lateral and longitudinal control of the vehicle, in a limited number of directions, albeit in different settings. However, although parking a vehicle involves more technically challenging manoeuvres, these are in more confined spaces, which requires greater spatial awareness, while also negotiating the presence of other road users coming from multiple directions. Therefore, drivers may provide higher ratings of the Parking ADF simply because it takes over a more challenging aspect of the whole driving task. Alternatively, it could be related to the level of risk that drivers are willing to accept. Since there is less risk related to controlling the vehicle at a very low speed in a carpark than managing it in a complicated environment with fast cars in adjacent lanes, drivers may feel more comfortable giving control in those circumstances. Finally, Park Assist systems are fairly common in modern vehicles, which was the most commonly owned ADAS (45%) amongst our sample; therefore, there may be an element of familiarity that lead to higher ratings for the Parking ADF. However, it is important to note that across ADFs, the intention to use scores were typically above 3, indicating that most respondents were neutral or positive regarding their intention to use any of the four ADFs.

### 3.2. Age and Gender Differences in Intention to Use Scores

To compare the effect of age group (18–29, 30–39, 40–49, 50–59, 60+ years) on the intention to use scores, we conducted four one-way, between-participant ANOVAs, one for each ADF type. Table 5 displays Welch’s F statistics, which indicate a significant effect of age group on Intention to use scores, across all four ADFs, with the intention to use ADFs decreasing with increasing age. Across all ADFs, the 30–39 age group had the highest mean intention to use score, followed by the 18–29 age group. In contrast, the 60+ age group had the lowest mean intention to use score across all ADFs. Results from the Games–Howell post hoc comparisons showed that across all ADFs, the means of all combinations of age groups differed significantly, except between the 18–29 and 30–39 age groups (Table 6). The estimated omega squared (ω^2^) for all ADFs was below 0.05, indicating that less than 5% of the total variation in the average intention to use score is attributable to differences between the age groups. These findings are in line with previous research, for example, Smith and Anderson (2017), who found that those under the age of 50 have a greater interest in riding in an autonomous vehicle than those aged 50 and older. The current research extends these findings to function-specific automated driving.

To compare the effect of gender (Male, Female) on the intention to use scores, we conducted four one-way, between-participant ANOVAs, one for each ADF type. The results presented in Table 5 indicate a significant effect of gender on intentions to use some ADFs, where males had higher intention to use scores for Motorway, Traffic Jam, and Urban ADFs. However, there was no difference between the two groups for the Parking ADF. The estimated omega squared (ω^2^) for all ADFs was below 0.01, indicating that less than 1% of the total variation in the average intention to use score is attributable to differences between the genders. These results are in line with those of Lee et al. (2019), who showed that gender was a significant predictor of acceptance of all ADAS investigated (Forward Collision Warning, Lane-Departure Warning, Lane-Keeping Assist, Adaptive Cruise Control) except Active Park Assist. This could be due to a number of factors. First, both genders most likely view parking as requiring a very different skillset, compared to other driving manoeuvres, which could explain the similarly high ratings for the Parking ADF. Second, females’ scepticism towards automated vehicles [18] may not extend to the Parking ADF, since parking could be considered relatively low risk in terms of injury or death, compared to the tasks the managed by the other ADFs. However, it is hard to know which of these two potential factors dominates, so more research is needed to better understand these gender differences.

### 3.3. The Effect of Experience with ADAS on Intention to Use Scores

We conducted a series of one-way ANOVAs (Welch’s F) to compare the mean intention to use scores for each ADF, based on whether respondents had experience with the respective ADAS (i.e., “Have it” vs. “Don’t have it”). Results shown in Table 7 indicate that across all ADFs, and for all ADAS, respondents who reported having that particular ADAS had significantly higher mean intention to use scores than those who did not. These differences are most noticeable for the Motorway, Traffic Jam, and Urban Road ADFs. However, these results do not mean that those who reported not owning a particular ADAS do not intend to use ADFs, as all the mean intention to use scores were still above 3 (or “Neutral” on the response scale). Overall, these results are in line with and extend those of [32], who showed that drivers who had ADAS in their own car were more comfortable with higher levels of automation than those who did not have these technologies in their cars. Our results go further to show that ADAS ownership is also associated with greater intention to use individual ADFs.

### 3.4. The Effect of Country on Intention to Use Scores

For the analysis of country differences, we calculated the Spearman rank-order correlation coefficients (*ρ*) between a country’s GDP per capita (WHO, 2018) and its mean intention to use score. These were also calculated between the country’s estimated number of road deaths per 100,000 population (WHO, 2018) and its mean intention to use score. The three countries with the highest GDP per capita were the USA (US$62,886), Sweden (US$54,651), and Finland (US$50,175). In contrast, the three countries with the lowest GDP per capita were India (US$2009), Indonesia (US$3893), and South Africa (US$6374). The three countries with the highest annual road deaths per 100,000 population were South Africa (25.9), India (22.6), and Brazil (19.7). In contrast, the countries with the three lowest road deaths per 100,000 population were Sweden (2.8), UK (3.1), and Germany, Japan, and Spain (all 4.1).

**Figure 1:**
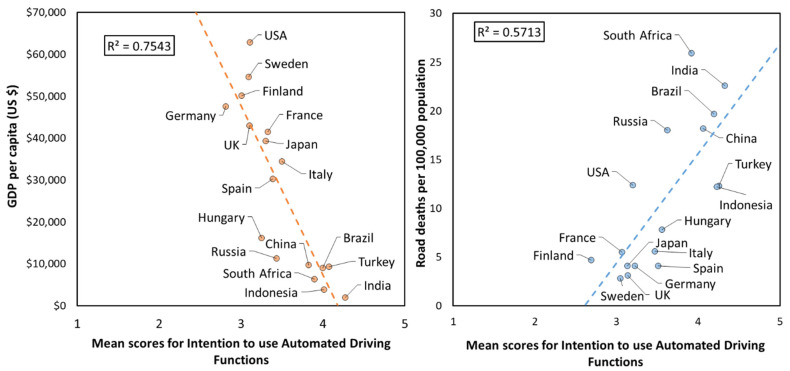
Correlation between 17 countries’ overall mean scores for intention to use Automated Driving Functions (ADFs) (combined) and their respective GDP per capita (**left**), and estimated road deaths per 100,000 population (**right** [33]).

There was a significant negative correlation between a country’s socio-economic status (GDP per capita) and the overall intention to use ADFs (*ρ* = −0.912, *p* < 0.0001, *n* = 17; Figure 1). On average, respondents from higher-GDP countries were more neutral regarding their intention to use ADFs, compared to those from lower-GDP countries, who tended to have higher intention to use scores. This pattern was similar when considering the ADFs separately, where there was a significant negative correlation between GDP and intention to use the Motorway (*ρ* = −0.914, *p* < 0.0001, *n* = 17), Traffic Jam, (*ρ* = −0.922, *p* < 0.0001, *n* = 17), Urban Roads (*ρ* = −0.870, *p* < 0.0001, *n* = 17), and Parking (*ρ* = −0.946, *p* < 0.0001, *n* = 17) ADFs.

There was a significant positive correlation between a country’s estimated number of road deaths per 100,000 population and the overall intention to use ADFs (*ρ* = 0.735, *p* < 0.001, *n* = 17), where countries with higher estimated road deaths tended to have higher intention to use scores. As with GDP, this pattern was similar for the different ADFs. There were significant positive correlations for the Motorway (*ρ* = 0.717, *p* < 0.001, *n* = 17), Traffic Jam, (*ρ* = 0.732, *p* < 0.001, *n* = 17), Urban Roads (*ρ* = 0.708, *p* < 0.001, *n* = 17), and Parking (*ρ* = 0.744, *p* < 0.001, *n* = 17) ADFs.

In high-GDP countries, as automated driving technologies start to become prevalent, the perceived risk associated with their use becomes more salient, leading to increased societal sensitivity to risks associated with use of these systems, resulting in a reduced intention to use. On the other hand, countries with low GDP are also often countries with higher road casualty rates (Figure 1). Higher exposure to road hazards can lead to risk normalisation, such that the consequences of crashes are underestimated by individuals [38]. Therefore, residents of countries with poor road safety records may not judge the possible risks associated with using an ADF as significant as that of driving without an ADF. The fact that there was a similar relationship between GDP/read death rate and intention to use across all ADFs, suggests that intention to use may be more intricately linked to individual and societal factors (i.e., age, gender, and country of residence) than the capabilities of the individual ADFs.

#### 3.4.1. The Effect of Age Group on Intention to Use across Different Countries

To compare the intention to use scores between age groups across the different countries, for each ADF, we conducted a series of one-way ANOVAs (Welch’s F). Table 8 shows that the intention to use scores for each ADF were generally higher for younger age groups and lower for older age groups across the 17 countries. However, this trend was only significant for a few countries. For example, in the USA, Sweden, India, and the UK, the mean intention to use scores decreased significantly with age, across all ADFs, which follows the general trend that younger individuals are more likely use technology (i.e., smartphones, tablet computers, and social media) compared to older individuals [39]. However, for some countries, while there was a statistically significant effect of age group on the intention use scores, the trend was not in line with the majority of other countries. For example, in China, for all ADFs except Parking, the 30–39 and 50–59 age groups had the highest intention to use scores, while the 18–29 age group had the lowest mean intention to use score, which contrasts with the trend in the majority of the other countries. This deviation is difficult to explain, though it could be that those in the 18–29 age group are more aware of the capabilities and limitations of the ADFs and may have less disposable income, compared to those in the older age groups. However, more research is needed to understand these intra-country differences.

Previously we showed that intention to use ADFs decreases with age, and that this pattern follows more general trends of technology acceptance and use. We also showed that intention to use is associated with lower GDP and higher road death rates, explained by differences in societal sensitivity to risks. Table 8 shows that, overall, the intention to use scores were highest among younger age groups in low-GDP countries, and lowest among older age groups in high-GDP countries. Therefore, while the effect of age on intention to use ADFs generally holds true across countries, the range may depend on the individual country’s sensitivity to risks.

#### 3.4.2. The Effect of Gender on Intention to Use across Different Countries

We conducted a series of one-way ANOVAs (Welch’s F) to compare the difference in the mean intention to use scores for males and females, for each country, and ADF (Table 9). Although the overall results show that males have a higher intention to use ADFs than females (except Parking ADF); this pattern is not consistent when considering individual countries, as there are certain countries for which this trend is more pronounced or reversed, for different ADFs. For example, males in the USA have significantly higher intention to use Motorway ADFs than females, but not the other ADFs. Similarly, males in Germany have significantly higher intention to use Traffic Jam ADFs than females, and males in Sweden have significantly higher intention to use Urban ADFs than females. However, females in India are significantly more likely to use Parking and Traffic Jam ADFs than males.

Overall, gender differences seem to be concentrated around countries with low road death rates. However, since countries with higher road death rates had higher mean intention to use scores overall, it could be that a lack of gender differences was due to a ceiling effect in the scores in those countries. In other words, since both genders already had high intention to use scores, it reduced the potential for there to be a difference between genders.

#### 3.4.3. The Effect of Experience with ADAS on Intention to Use across Different Countries

We conducted a series of one-way ANOVAs to compare the difference in the mean intention to use scores for those who stated they did/did not have ADAS in their vehicles. We used Self-Park Assist (SPA) experience to explore intention to use the Parking ADF and experience with Adaptive Cruise Control (ACC) for the other ADFs. Table 10 shows that the effect of experience with ACC on intention to use different ADFs clearly differs between countries. There seem to be fewer differences within lower-GDP countries for those who do and do not have ADAS. Overall, however, those with ACC consistently had higher intention to use scores than those without. The notable exception is Finland, where respondents who had ACC reported significantly (*p* < 0.01) lower intention to use scores for Motorway ADFs (M = 2.49) compared to those who did not (M = 3.03).

Regarding the Parking ADF, there was no overall effect of experience with ADAS on intention to use across countries, except for India and the UK, where owning a Self-Park Assist was associated with higher intention to use Parking ADFs. Overall, the effect of experience with ADAS appears to be concentrated around high-GDP countries and for the Motorway, Traffic Jam, and Urban ADFs. Although one might expect this to be because respondents from low-GDP countries have not had direct experience with ADAS, Table 10 shows that there was a higher proportion of respondents from low-GDP countries who reported having ADAS, compared to respondents from high-GDP countries. Since the distributions of annual mileage, household income, and education level amongst our sample were similar across countries, it cannot be said that higher intention to use among respondents from low-GDP countries is associated with a lack of experience with ADAS, or any major differences in socio-demographic variables. This higher intention to use score from respondents in low-GDP countries reflects a broadly more positive view of the potential benefits of automation, such as its contributions to improving road safety, and increasing mobility [38]. That said, future research should also consider intra-country differences in perceptions towards ADFs, as developed and underdeveloped areas in the same country may vary greatly.

## 4. Conclusions

Several studies have investigated the acceptance of conditionally automated cars (CACs). However, there is limited knowledge of drivers’ intention to use CACs in specific ODDs and how factors such as age, gender, experience with ADAS, and country of residence influence these. In the present survey-based study, we asked 18,631 respondents, from 17 countries across the globe, about their intention to use automated driving functions (ADFs) in Motorway, Traffic Jam, Urban, and Parking ODDs.

We found that the intention to use scores were high across all ADFs, and particularly for Parking. However, trends varied widely, based on respondents’ age, gender, experience with ADAS, and country of residence. For example, we found a significant negative correlation between age and the intention to use ADFs across all ODDs, but this effect was more pronounced in high-GDP countries. In addition, we found that males had a greater intention to use ADFs compared to females, except in Parking settings, where there was no difference. Importantly, however, we also showed that the effect of gender on intention to use ADFs was generally stronger in countries with middle-to-high-income and low road-related death rates, but that this varied between ADFs. The results also showed that ownership of any number, or type, of ADAS was associated with higher intention to use all ADFs, and analysis of cross-national differences showed that this was particularly prevalent in middle-to-high-income countries. Finally, we also showed that there was a significant negative correlation between a country’s Gross Domestic Product, per capita, and intention to use ADFs, regardless of ODDs, with a significant positive correlation between a country’s estimated road death rate and a resident’s intention to use any ADFs. Results also showed a significant negative correlation between GDP and the intention to use ADFs. These results highlight the relevance of cross-national and socio-demographic differences when investigating acceptance of potential future users of CACs, and their role in the development and deployment of CACs.

Our results suggest that residents from less developed countries might be more willing to adopt CACs, which could reflect respondents’ hope that these technologies will improve road safety. On the other hand, personal automated vehicles have often been associated with an increase in the number of vehicle kilometres travelled [40], and large-scale adoption of CACs in low-GDP countries, which already struggle with problems associated with increased motorisation rates, could contribute further to this issue. Overall, the value of shared CACs or automated public transport systems could be one way of providing better mobility, also managing the number of vehicles deployed, and the vehicle kilometres travelled [41]. To support the large-scale deployment of CACs, city authorities, city planners, and operators need to work together to ensure that the necessary infrastructure is put in place.

Our results do not highlight a specific need to develop different strategies for the deployment of different ADFs. However, development and deployment strategies for CACs may need to be tailored to different markets and customers with different socio-economic profiles, to promote the uptake and safe use of this technology. For example, in markets where the intention to use CACs is low (countries with high GDP, and low road casualty rate), more emphasis should be placed on communicating the safety benefits of the technology, especially to older cohorts. On the other hand, in markets where the intention to use CACs is high (countries with low GDP and high road casualty rate), it may be necessary to develop a pathway for accessing ADFs that avoids cost being the barrier to adoption (e.g., government-funded grants, vis-à-vis electric vehicles). Finally, given the enthusiastic view of CACs in these countries, it may be prudent to communicate the realities of the limitations of the technologies to avoid potential misuse due to inflated expectations, especially amongst younger cohorts.

The major limitation of this, and most, online surveys in this context is that respondents did not have the opportunity to physically experience these systems. This limitation may bias respondents’ views, and lead to inaccurate, or inflated, expectations about the capabilities of ADFs. Since conditionally automated vehicles, including their various ADFs, are not currently widely available, it was not possible to counteract this shortcoming. However, this concern was lessened in the current study, as our focus was to investigate the intention to use, rather than overall system acceptance.

The current research provides a limited, but detailed, account of the influence and interaction of a small number of factors thought to influence drivers’ intention to use CACs. Future research on the acceptance of specific ADFs should investigate the effect of other factors contributing to technology acceptance, such as user satisfaction and trust, system performance, and effort expectancy [5], the modal share of an individual’s travel behaviour [42], and also an individual’s previous experience of traffic accidents when using ADAS. However, for these approaches to be meaningful, future research needs to investigate detailed use cases, with the aid of physical demonstrations or more naturalistic, longer-term use, as experience is known to increase acceptance of automated vehicles [43,44].

## 5. Acknowledgments

Responsibility for the information and views set out in this publication lies entirely with the authors.

## Figures and Tables

**Table 1:** System descriptions for Automated Driving Functions (ADFs) in motorways, traffic jams, urban areas, and parking settings.

Operational Design Domain (ODD)	Automated Driving Function (ADF) Description	Questionnaire Item Measuring Behavioural Intention
Motorway	A conditionally automated car on motorways stays in the lane, follows the vehicle in front and overtakes slower vehicles at a maximum speed of up to 130 km/h.	*“I plan to use a conditionally automated car on motorways once it becomes available.”*
Traffic Jam	On congested motorways, a conditionally automated car takes over the driving in a traffic jam up to 60 km/h, identifies slower vehicles in front and changes the lane to overtake slower vehicles or to exit the motorway.	*“I plan to use a conditionally automated car on congested motorways once it becomes available.”*
Urban	A conditionally automated car on urban roads follows the lane, accelerates, decelerates, identifies, and overtakes other road users, including pedestrians and cyclists. It can also handle crossings and automatically turns right or left.	*“I plan to use a conditionally automated car on urban roads once it becomes available.”*
Parking	A conditionally automated car in parking situations overtakes the parking into and out of garages and driveways. The driver can either be inside or outside the vehicle. The parking manoeuvre does not have to be monitored by the driver.	*“I plan to use a conditionally automated car in parking situations once it becomes available.”*

**Table 2:** Original and recoded items for ADAS experience.

Original Items for ADAS Experience	Recoded Items for Analysis
“I have it and I use it”	“Have it”
“I have it but I don’t use it”	
“Don’t know if I have it”	“Don’t have it”
“I don’t have it but I would use it”	
“I don’t have it and I would not use it”	

**Table 3:** ADAS descriptions given to the survey respondents.

ADAS	Description
Automated Emergency Braking (AEB)	A system that automatically brakes the vehicle when an impending collision is detected.
Forward Collision Warning (FCW)	A system that provides warnings for potential collisions with the vehicle in front.
Blind Spot Monitoring (BSM)	A system that monitors the driver’s left and right blind spots for other vehicles. Often, drivers receive a visual or audio alert whenever a vehicle is present.
Drowsy Driver Detection (DDD)	A system that detects driver drowsiness.
Lane Departure Warning (LDW)	A system that provides assistance with lane-keeping by sounding warnings when the vehicle travels outside the current lane’s markings/boundaries of the current lane.
Lane Keeping Assistance (LKA)	A system that helps the driver to avoid inadvertently moving out of a lane.
Adaptive Cruise Control (ACC)	A system that maintains vehicle speed while in cruise control mode, but automatically slows down or speeds up to keep a driver-selected distance from a vehicle ahead.
Parking Assist (PA)	Radar, beeps, or camera view. The driver is in the car during the parking manoeuvre.
Self-Parking Assist (SPA)	A system that controls the vehicle for parallel or reverse parking. The system may control both steering and the throttle, or only control the steering (the driver presses the brake and throttle) during the parking manoeuvre. The driver is in the car during the parking manoeuvre.

**Table 4:** Sample size for each country, segmented by age group and gender.

	Total (*n*)	18–29 Years		30–39 Years		40–49 Years		50–59 Years		60+ Years	
		Male	Female	Male	Female	Male	Female	Male	Female	Male	Female
**Brazil**	1057	141	161	162	190	103	101	79	54	37	27
**China**	1004	170	132	156	143	104	130	54	67	22	25
**Finland**	1021	22	50	75	54	150	94	208	120	145	103
**France**	1164	104	130	94	164	143	146	97	108	116	59
**Germany**	1133	114	116	95	117	116	118	130	137	107	83
**Hungary**	1146	109	156	108	155	151	113	101	80	94	79
**India**	1054	181	170	196	212	90	73	35	34	38	24
**Indonesia**	1059	146	203	191	185	144	81	42	36	16	15
**Italy**	1186	103	125	130	137	165	123	119	147	76	57
**Japan**	1074	47	107	106	124	127	121	132	128	118	62
**Russia**	1079	133	140	151	231	113	119	67	66	34	24
**South Africa**	1070	206	223	128	148	97	101	51	57	27	32
**Spain**	1074	77	117	129	142	162	116	114	113	60	42
**Sweden**	1177	146	155	128	112	105	95	110	137	119	67
**Turkey**	1060	156	144	164	200	124	103	52	64	35	17
**UK**	1217	129	151	148	173	134	132	96	98	91	64
**USA**	1056	135	98	86	133	90	109	104	110	87	97
**Total**	**18,631**	**2119**	**2378**	**2247**	**2620**	**2118**	**1875**	**1591**	**1556**	**1222**	**877**

**Table 5:** Mean (M), standard deviation (SD), and ANOVA test results for intention to use different ADFs, by age group and gender.

		Motorways		Traffic Jam		Urban		Parking	
		M	SD	M	SD	M	SD	M	SD
**Age**	18–29	3.63	1.1	3.52	1.12	3.58	1.11	3.82	1.01
	30–39	3.7	1.08	3.61	1.15	3.63	1.12	3.89	0.99
	40–49	3.43	1.17	3.43	1.16	3.39	1.15	3.66	1.1
	50–59	3.18	1.2	3.21	1.15	3.12	1.19	3.52	1.16
	60+	2.89	1.25	2.92	1.23	2.91	1.26	3.28	1.22
	*Welch’s F*	F (4,1992.71) = 59.946, ω^2^ = 0.04 ***		F (4,2001.20) = 38.649, ω^2^ = 0.03 ***		F (4,1999.64) = 50.886, ω^2^ = 0.03 ***		F (4,1924.19) = 34.244, ω^2^ = 0.03 ***	

**Gender**	Male	3.51	1.15	3.49	1.14	3.44	1.17	3.7	1.09
	Female	3.38	1.2	3.31	1.2	3.35	1.19	3.7	1.1
	*Welch’s F*	F (4,4608.97) = 14.154, ω^2^ < 0.01 ***		F (1,4595.34) = 25.739, ω^2^ < 0.01 ***		F (1,4621) = 6.322, ω^2^ < 0.01 **		F (1,4625.772) = 0.014, ω^2^ < 0.01	

** *p* < 0.01, *** *p* < 0.001.

**Table 6:** Post-hoc results for the intention to use scores by age group and ADF.

Age Group	18–29	30–39	40–49	50–59	60+
18–29	x				
30–39	ns	x			
40–49	M ** U ** P **	M ** T ** U ** P **	x		
50–59	M ** T ** U ** P **	M ** T ** U ** P **	M ** T ** U ** P **	x	
60+	M ** T ** U ** P **	M ** T ** U ** P **	M ** T ** U ** P **	M ** T ** U ** P **	x

M: Motorway, T: Traffic Jam, U: Urban, P: Parking, ns: not significant; ** *p* < 0.01.

**Table 7:** Sample size (*n*), mean (M), standard deviation (SD), and independent t-test results for intention to use different ADFs by experience with different ADAS. *** *p* < 0.001.

		Motorway ADF			Traffic Jam ADF			Urban ADF			Parking ADF		
		*n*	M	SD	*n*	M	SD	*n*	M	SD	*n*	M	SD
**AEB**	**Don’t have it**	2981	3.27	1.19	3006	3.23	1.18	2938	3.22	1.18	2975	3.60	1.12
	**Have it**	1452	3.83	1.06	1431	3.77	1.08	1491	3.79	1.07	1440	3.95	1.00
	*Welch’s F*	−15.758 ***			−15.013 ***			−16.130 ***			−10.385 ***		
**FCW**	**Don’t have it**	3182	3.29	1.18	3202	3.25	1.17	3199	3.24	1.18	3198	3.62	1.11
	**Have it**	1251	3.86	1.08	1240	3.79	1.10	1236	3.84	1.05	1212	3.97	1.00
	*Welch’s F*	−15.383 ***			−14.334 ***			−16.523 ***			−10.217 ***		
**BSM**	**Don’t have it**	3204	3.28	1.19	3225	3.25	1.18	3244	3.24	1.18	3239	3.60	1.11
	**Have it**	1232	3.89	1.02	1218	3.81	1.08	1193	3.86	1.04	1175	4.01	0.98
	*Welch’s F*	−16.902 ***			−15.024 ***			−16.876 ***			−11.622 ***		
**DDD**	**Don’t have it**	3550	3.33	1.17	3579	3.29	1.17	3633	3.29	1.17	3618	3.64	1.10
	**Have it**	882	3.95	1.08	854	3.88	1.10	795	3.95	1.06	795	4.04	0.99
	*Welch’s F*	−14.951 ***			−14.063 ***			−15.539 ***			−10.016 ***		
**LDW**	**Don’t have it**	3213	3.31	1.18	3254	3.26	1.18	3271	3.26	1.17	3232	3.63	1.10
	**Have it**	1219	3.84	1.08	1191	3.79	1.10	1160	3.84	1.08	1177	3.94	1.02
	*Welch’s F*	−14.395 ***			−13.882 ***			−15.357 ***			−8.893 ***		
**PA**	**Don’t have it**	2387	3.26	1.17	2413	3.22	1.18	2468	3.19	1.17	2489	3.59	1.10
	**Have it**	2046	3.66	1.16	2021	3.62	1.14	1967	3.68	1.13	1931	3.87	1.06
	*Welch’s F*	−11.403 ***			−11.445 ***			−14.010 ***			−8.578 ***		
**LKA**	**Don’t have it**	3362	3.31	1.18	3367	3.28	1.18	3322	3.27	1.18	3365	3.62	1.10
	**Have it**	1070	3.90	1.07	1070	3.79	1.11	1104	3.84	1.07	1052	4.00	0.99
	*Welch’s F*	−15.225 ***			−12.799 ***			−14.919 ***			−10.381 ***		
**ACC**	**Don’t have it**	2769	3.29	1.17	2804	3.23	1.19	2818	3.24	1.17	2761	3.60	1.11
	**Have it**	1671	3.71	1.15	1635	3.69	1.12	1617	3.69	1.14	1658	3.89	1.04
	*Welch’s F*	−11.748 ***			−12.909 ***			−12.429 ***			−8.609 ***		
**SPA**	**Don’t have it**	3290	3.28	1.19	3274	3.26	1.19	3269	3.23	1.18	3388	3.63	1.11
	**Have it**	1145	3.93	1.02	1174	3.80	1.07	1176	3.90	1.01	1037	3.99	0.97
	*Welch’s F*	−17.823 ***			−14.173 ***			−18.739 ***			−10.015 ***		

**Table 8:** Mean intention to use scores across countries (ordered from lowest to highest GDP; [33]) and for different ADFs and age groups. See below figure for colour scale. * *p* < 0.05, ** *p* < 0.01, *** *p* < 0.001

	Motorway						Traffic Jam						Urban						Parking					
	18–29	30–39	40–49	50–59	60+	*p*	18–29	30–39	40–49	50–59	60+	*p*	18–29	30–39	40–49	50–59	60+	*p*	18–29	30–39	40–49	50–59	60+	*p*
India	4.33	4.62	4.24	4.15	4.25	**	3.93	4.46	4.35	3.62	3.83	***	4.20	4.39	4.20	3.86	4.06		4.10	4.49	4.25	3.91	3.69	**
Indonesia	4.11	4.23	4.04	4.04	2.80		3.79	4.06	3.89	3.83	4.00		3.73	4.00	4.03	3.44	3.80		4.15	4.18	4.03	4.46	4.18	
South Africa	3.83	3.83	4.02	4.00	3.56		3.95	3.65	3.92	3.39	3.38	*	3.94	3.87	3.73	3.69	3.44		4.23	4.21	3.84	4.17	3.78	
Brazil	3.92	3.98	3.85	3.97	3.86		3.89	4.12	3.90	3.94	3.71		4.01	4.09	3.66	3.61	3.86		4.23	4.35	3.96	4.25	3.67	
Turkey	4.23	4.09	4.02	4.04	4.33		3.64	4.20	3.95	3.83	3.92	*	4.15	4.14	4.24	4.09	4.25		4.08	4.07	4.14	4.03	4.25	
China	3.36	3.92	3.81	4.03	3.75	***	3.32	3.70	3.75	3.81	4.00	**	3.77	4.25	4.02	4.11	4.00	**	3.93	3.98	3.95	3.61	3.86	
Russia	3.52	3.28	3.42	3.24	3.35		3.79	3.47	3.36	3.06	3.00	**	3.08	3.42	3.16	3.19	2.93		3.77	3.74	3.76	3.35	3.33	
Hungary	3.20	3.36	2.97	3.06	3.06		3.27	2.83	3.11	3.27	3.28		3.16	3.07	3.11	3.19	3.13		3.69	3.61	3.55	3.46	3.51	
Spain	3.55	3.59	3.35	2.95	2.83	**	3.34	3.42	3.30	3.23	3.13		3.35	3.32	3.33	3.06	3.19		3.75	3.76	3.59	3.74	3.33	
Italy	3.68	3.49	3.56	3.34	3.03		3.58	3.45	3.52	3.38	2.94		3.50	3.44	3.54	3.36	3.26		3.78	3.91	3.76	3.55	3.24	
Japan	2.98	3.43	3.23	3.37	2.98		3.11	3.26	3.01	3.33	3.00		3.16	3.36	3.29	3.28	3.45		3.37	3.51	3.67	3.47	3.49	
France	3.52	3.77	3.59	2.96	2.52		3.48	3.40	3.32	3.28	2.88		3.33	3.63	3.34	2.76	2.55	***	3.67	3.55	3.63	3.23	3.26	
UK	3.33	3.55	3.25	2.60	2.22	***	3.21	3.35	2.97	2.64	2.15	***	3.26	3.24	2.87	2.61	2.21	***	3.33	3.65	3.46	3.35	2.90	*
Germany	2.67	2.70	2.58	2.61	2.45		3.25	2.75	3.03	2.90	2.55		3.00	2.59	2.55	2.61	2.15	**	3.28	3.52	3.00	3.10	2.72	**
Finland	2.00	3.32	3.06	2.80	2.79		2.14	2.69	3.11	2.99	2.71		3.06	3.05	2.96	2.76	3.05		3.58	3.52	3.32	3.19	3.15	
Sweden	3.42	3.02	3.04	2.72	2.63	***	3.16	3.20	3.33	3.17	2.51	**	3.20	3.02	2.71	2.77	2.24	**	3.53	3.67	3.41	3.33	3.08	
USA	3.40	3.30	2.77	2.94	2.64	**	3.17	3.46	3.14	2.61	2.45	***	3.52	3.27	2.96	2.85	2.37	***	3.53	3.60	3.22	3.45	3.07	
	1	Low intention to use																						
	5	High intention to use																						

**Table 9:** Differences in the mean intention to use scores for males vs. females across the 17 countries (ordered from lowest to highest GDP; [33]). A positive number highlighted green indicates that males had a significantly higher intention to use score than females. A negative number highlighted orange indicates that females had a significantly higher intention to use score than males.

	“Male”–“Female”			
	Motorway	Traffic Jam	Urban	Parking
India	0.03	−0.27 *	−0.03	−0.45 ***
Indonesia	−0.05	−0.05	0.05	−0.04
S. Africa	0.2	0.46 ***	0.13	0.05
Brazil	0.1	0.05	−0.04	−0.1
Turkey	−0.33 *	−0.26	−0.31 *	−0.2
China	−0.07	−0.14	0.06	0.04
Russia	0.08	0.18	−0.13	0.09
Hungary	0.02	0.08	−0.1	−0.16
Spain	0.29 *	0.36 **	0.38 **	−0.09
Italy	0.26 *	0.35 **	0.08	0.31 *
Japan	0.28 *	0.36 **	0.29 *	0.11
France	0.26 *	0.17	−0.05	0.24
UK	0.22	0.32 **	0.07	−0.03
Germany	0.22	0.68 ***	0.11	0.36 **
Finland	0.18	0.33 *	0.19	−0.23
Sweden	0.18	0.18	0.63 ***	−0.06
USA	0.53 ***	0.29	0.17	0.08

* *p* < 0.05, ** *p* < 0.01, *** *p* < 0.001.

**Table 10:** Differences in the mean intention to use scores across countries (ordered from lowest to highest GDP; [33]) and for different ADFs between drivers who do and do not have ADAS (Self-Park Assist; SPA) for the Parking ADF and Adaptive Cruise Control (ACC) for all other ADFs. A positive number highlighted green indicates that males had a significantly higher intention to use score than females. A negative number highlighted orange indicates that females had a significantly higher intention to use score than males.

	“Have ACC”–“Do Not Have ACC”				“Have SPA”–“Do Not Have SPA”	
	Motorway	Traffic Jam	Urban	% Have ACC	Parking	% Have SPA
India	0.42 **	0.69 ***	0.31 *	60%	0.34 **	60%
Indonesia	0.31 *	0.34 *	0.32 *	45%	0.07	44%
S. Africa	0.23	0.16	0.25	40%	0.20	20%
Brazil	0.21	0.17	0.39 **	32%	0.21	32%
Turkey	0.12	−0.01	0.10	59%	0.13	43%
China	0.25	0.06	0.17	61%	−0.13	60%
Russia	0.14	0.22	0.34	37%	0.24	19%
Hungary	0.44 **	0.42 **	0.38 *	20%	−0.03	12%
Spain	0.58 ***	0.38 **	0.34 **	44%	0.25	24%
Italy	0.29	0.41 *	0.03	23%	−0.06	18%
Japan	−0.09	0.51 **	0.32 *	21%	−0.09	13%
France	0.05	0.39 **	0.35 **	45%	−0.27	16%
UK	0.46 ***	0.45 ***	0.45 ***	33%	0.61 ***	21%
Germany	0.22	0.56 ***	0.70 ***	24%	0.27	19%
Finland	−0.55 **	0.12	0.19	20%	0.22	8%
Sweden	0.38 **	0.06	0.10	33%	0.21	17%
USA	0.33 *	0.4 **	0.20	39%	0.32	14%

* *p* < 0.05, ** *p* < 0.01, *** *p* < 0.001.

## Data Availability

The full dataset used in this study is hosted on Zenodo, an open-access repository developed under the European OpenAIRE program and operated by CERN: https://zenodo.org/record/5255950#.YWb8r8DRYWM.

## References

[1] Buckley L., Kaye S.A., Pradhan A.K. (2018). Psychosocial factors associated with intended use of automated vehicles: A simulated driving study. Accid. Anal. Prev..

[2] Dixon G., Hart P.S., Clarke C., O’Donnell N.H., Hmielowski J. (2020). What drives support for self-driving car technology in the United States?. J. Risk Res..

[3] Liu P., Yang R., Xu Z. (2019). Public acceptance of fully automated driving: Effects of social trust and risk/benefit perceptions. Risk Anal..

[4] Becker F., Axhausen K.W. (2017). Literature review on surveys investigating the acceptance of automated vehicles. Transportation.

[5] Nordhoff S., Louw T., Innamaa S., Lehtonen E., Beuster A., Torrao G., Bjorvatn A., Kessel T., Malin F., Happee R. (2020). Using the UTAUT2 model to explain public acceptance of conditionally automated (L3) cars: A questionnaire study among 9118 car drivers from eight European countries. Transp. Res. Part F Traffic Psychol. Behav..

[6] JD Power (2020). J.D. Power, Survey Monkey Find Most Consumers Still Say ‘Thanks, but No Thanks’ to Automakers’ Future Self-Driving and Electric Vehicle Offerings. http://www.jdpower.com/pr-id/2020003.

[7] Smith A., Anderson M. (2017). Automation in Everyday Life.

[8] SAE International (2021). Taxonomy and Definitions for Terms Related to Driving Automation Systems for On-Road Motor Vehicles. https://www.sae.org/standards/content/j3016_202104/.

[9] Son J., Park M., Park B.B. (2015). The effect of age, gender and roadway environment on the acceptance and effectiveness of Advanced Driver Assistance Systems. Transp. Res. Part F Traffic Psychol. Behav..

[10] Warshaw P.R., Davis F.D. (1985). Disentangling behavioral intention and behavioral expectation. J. Exp. Soc. Psychol..

[11] Venkatesh V., Morris M.G., Davis G.B., Davis F.D. (2003). User acceptance of information technology: Toward a unified view. MIS Q..

[12] Madigan R., Louw T., Wilbrink M., Schieben A., Merat N. (2017). What influences the decision to use automated public transport? Using UTAUT to understand public acceptance of automated road transport systems. Transp. Res. Part F Traffic Psychol. Behav..

[13] Schoettle B., Sivak M. (2015). Motorists’ Preferences for Different Levels of Vehicle Automation.

[14] Krueger R., Rashidi T.H., Rose J.M. (2016). Preferences for shared autonomous vehicles. Transp. Res. Part C Emerg. Technol..

[15] Molin E., Mokhtarian P., Kroesen M. (2016). Multimodal travel groups and attitudes: A latent class cluster analysis of Dutch travelers. Transp. Res. Part A Policy Pract..

[16] Liljamo T., Liimatainen H., Pöllänen M. (2018). Attitudes and concerns on automated vehicles. Transp. Res. Part F Traffic Psychol. Behav..

[17] Zmud J.P., Sener I.N. (2017). Towards an understanding of the travel behavior impact of autonomous vehicles. Transp. Res. Procedia.

[18] Payre W., Cestac J., Delhomme P. (2014). Intention to use a fully automated car: Attitudes and a priori acceptability. Transp. Res. Part F Traffic Psychol. Behav..

[19] Zhang R. (2020). Understanding Customers’ Attitude and Intention to Use Driverless Cars.

[20] Venkatesh V., Morris M.G., Ackerman P.L. (2000). A longitudinal field investigation of gender differences in individual technology adoption decision-making processes. Organ. Behav. Hum. Decis. Process..

[21] Kyriakidis M., Happee R., de Winter J.C. (2015). Public opinion on automated driving: Results of an international questionnaire among 5000 respondents. Transp. Res. Part F Traffic Psychol. Behav..

[22] Silberg G., Manassa M., Everhart K., Subramanian D., Corley M., Fraser H., Sinha V. (2013). Self-Driving Cars: Are We Ready.

[23] Delle Site P., Filippi F., Giustiniani G. (2011). Users’ preferences towards innovative and conventional public transport. Procedia-Soc. Behav. Sci..

[24] Rödel C., Stadler S., Meschtscherjakov A., Tscheligi M. Towards autonomous cars: The effect of autonomy levels on acceptance and user experience. Proceedings of the 6th International Conference on Automotive User Interfaces and Interactive Vehicular Applications.

[25] Nordhoff S., De Winter J., Kyriakidis M., Van Arem B., Happee R. (2018). Acceptance of driverless vehicles: Results from a large cross-national questionnaire study. J. Adv. Transp..

[26] Lee C., Gershon P., Reimer B., Mehler B., Coughlin J. (2021). Consumer Knowledge and Acceptance of Driving Automation: Changes over Time and across Age Groups. Proceedings of the Human Factors and Ergonomics Society Annual Meeting.

[27] Jenness J.W., Lerner N.D., Mazor S., Osberg J.S., Tefft B.C. (2008). Use of advanced in-vehicle technology by young and older early adopters. Survey results on adaptive cruise control systems. Rep. No. DOT HS.

[28] Ng W.S., Acker A. (2018). Understanding Urban Travel Behaviour by Gender for Efficient and Equitable Transport Policies. International Transport Forum Discussion Paper; International Transport Forum, Paris, France. https://www.itf-oecd.org/sites/default/files/docs/urban-travel-behaviour-gender.pdf.

[29] Moriarty P., Honnery D. Determinants of urban travel in Australia. Proceedings of the 28th Australasian Transport Research Forum (ATRF).

[30] Best H., Lanzendorf M. (2005). Division of labour and gender differences in metropolitan car use: An empirical study in Cologne, Germany. J. Transp. Geogr..

[31] Boarnet M.G., Sarmiento S. (1998). Can land-use policy really affect travel behaviour? A study of the link between non-work travel and land-use characteristics. Urban Stud..

[32] Lee C., Seppelt B., Reimer B., Mehler B., Coughlin J.F. (2019). Acceptance of vehicle automation: Effects of demographic traits, technology experience and media exposure. Proceedings of the Human Factors and Ergonomics Society Annual Meeting (Vol. 63, No. 1, pp. 2066–2070).

[33] World Health Organization (2018). Global Status Report on Road Safety 2018: Summary (No. WHO/NMH/NVI/18.20).

[34] Frost & Sullivan (2018). Global Autonomous Driving Market Outlook 2018.

[35] Bazilinskyy P., Kyriakidis M., de Winter J. (2015). An international crowdsourcing study into people’s statements on fully automated driving. Procedia Manuf..

[36] Nordhoff S., de Winter J., Madigan R., Merat N., van Arem B., Happee R. (2018). User acceptance of automated shuttles in Berlin-Schöneberg: A questionnaire study. Transp. Res. Part F Traffic Psychol. Behav..

[37] Venkatesh V., Thong J.Y., Xu X. (2012). Consumer acceptance and use of information technology: Extending the unified theory of acceptance and use of technology. MIS Q..

[38] Lima M.L., Barnett J., Vala J. (2005). Risk Perception and Technological Development at a Societal Level. Risk Anal..

[39] Vogels E.A. (2019). Millennials Stand out for Their Technology Use, but Older Generations also Embrace Digital Life.

[40] Soteropoulos A., Berger M., Ciari F. (2019). Impacts of automated vehicles on travel behaviour and land use: An international review of modelling studies. Transp. Rev..

[41] Fagnant D.J., Kockelman K. (2015). Preparing a nation for autonomous vehicles: Opportunities, barriers and policy recommendations. Transp. Res. Part A Policy Pract..

[42] Lehtonen E., Malin F., Innamaa S., Nordhoff S., Louw T., Bjorvatn A., Merat N. (2021). Are multimodal travellers going to abandon sustainable travel for L3 automated vehicles?. Transp. Res. Interdiscip. Perspect..

[43] Dikmen M., Burns C.M. Autonomous driving in the real world: Experiences with tesla autopilot and summon. Proceedings of the 8th International Conference on Automotive User Interfaces and Interactive Vehicular Applications.

[44] Rahman M.M., Lesch M.F., Horrey W.J., Strawderman L. (2017). Assessing the utility of TAM, TPB, and UTAUT for advanced driver assistance systems. Accid. Anal. Prev..

